# Superior performance of Na_7_V_4_(P_2_O_7_)_4_PO_4_ in sodium ion batteries†

**DOI:** 10.1039/c8ra03682a

**Published:** 2018-06-11

**Authors:** Wenying Fang, Zhongxun An, Jiaqiang Xu, Hongbin Zhao, Jiujun Zhang

**Affiliations:** Shanghai Aowei Technology Development Co., Ltd. Shanghai 201203 PR China; School of Materials Science and Engineering, Shanghai University Shanghai 200444 PR China; Colllege of Science, Shanghai University Shanghai 200444 PR China; NRC Energy, Mining and Environment, National Research Council of Canada 4250 Wesbrook Mall Vancouver V6T 1W5 Canada

## Abstract

A novel synthetic method has been investigated to fabricate a 1D nanostructure Na_7_V_4_(P_2_O_7_)_4_PO_4_. Mixed polyanion materials with a well-defined 3D framework channel can improve the electrochemical performance of sodium reversible insertion/extraction reactions, and can be especially beneficial for high rate performance and cycling capability. It approaches an initial reversible electrochemical capacity of 92.0 mA h g^−1^ with a high discharge potential over 3.85 V (*vs.* Na/Na^+^) and good cycling properties with a capacity retention of 81.4% after 300 cycles at a 0.5C rate in sodium systems. Taking into consideration the superior electrochemical characteristics, the phase-pure composite is considered to have a promising high rate capability as well as being a high capacity electrode material for advanced energy storage applications.

## Abstract 2

A novel synthetic method has been investigated to fabricate a 1D nanostructure Na_7_V_4_(P_2_O_7_)_4_PO_4_.

## Graphical abstract

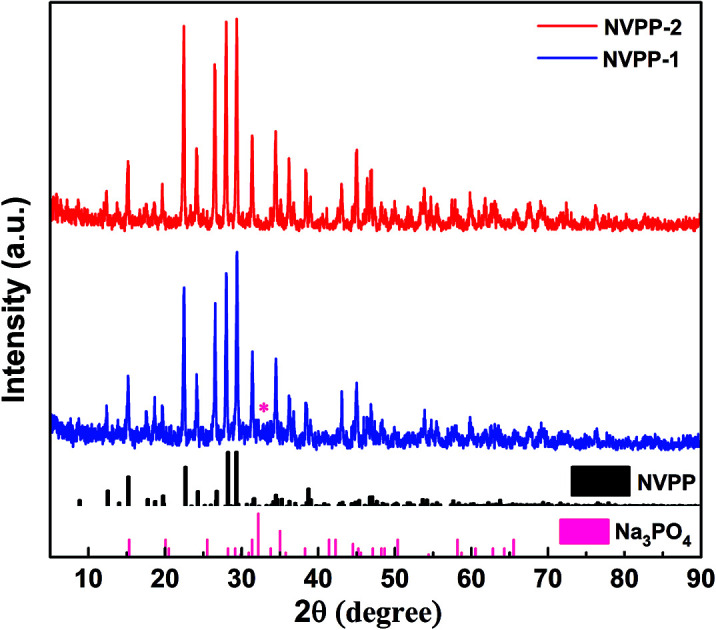

## 1. Introduction

Lithium ion batteries (LIBs) are obviously distinguished from many other rechargeable batteries as power sources for applications of electrical vehicles due to their superior power and energy densities.^1–3^ However, existing challenges, such as low distribution on the Earth, high cost, and the lack of high performance cathode materials have limited the development of advanced LIBs, especially in the generation of electrical vehicles and smart grids.^4,5^ Alternatively, sodium ion batteries (SIBs) are considered to be an attractive alternative for renewable large-scale energy storage devices and have had increasingly tremendous attention for their sustainable abundant reserves and low cost, as well as their similar electrochemical performance compared to LIBs.^6–8^ The primary restriction of the reaction kinetics and volume change in the process of electrochemical insertion/extraction is the larger ionic radius of Na^+^ compared to that of Li^+^ (1.02 Å *vs.* 0.76 Å). Therefore, there is an urgent desire to investigate large interstitial channel cathode electrode materials such as highly three-dimensional frameworks to cushion the volume expansion caused by rapid Na^+^ shuttling.^9,10^

In recent years, many considerable efforts have been made to develop novel cathode materials for rechargeable SIBs,^11–14^ for instance, Na_*x*_CoO_2_,^15^ Na_0.44_MnO_2_,^16^ NaFeO_2_,^17^ NaFePO_4_,^18^ Na_3_M_2_(PO_4_)_3_ (M = Ti, Fe)^19^ and Na_3_M_2_(PO_4_)_2_F.^20^ Among the various cathode materials for SIBs, a vanadium-based *ortho*-diphosphate Na_7_V_4_(P_2_O_7_)_4_PO_4_ (NVPP) with a high energy density and stable cycling performance exhibits a single-valued voltage plateau at 3.88 V, where the remarkable single plateau and cycle life originate from an immediate phase (a very shallow voltage step), and has attracted comprehensive attention due to its unique 3D structure with a central tetrahedron [PO_4_] sharing corners with four [VO_6_] octahedra in the (VP_2_O_7_)_4_PO_4_ unit, and each diphosphate group [P_2_O_7_] bridging the two adjacent [VO_6_] octahedra by sharing the corners. The interconnected (VP_2_O_7_)_4_PO_4_ units form a 3D framework with well-defined ionic channels for Na (de)insertion.^21^ Na ions occupy three different crystallographic positions, Na1, Na2, and Na3, through the open 3D framework enabling reversible sodium de/intercalation which is favorable for surprising electrochemical behavior. Lim *et al.*^21^ reported a vanadium-based *ortho*-diphosphate NVPP that exhibited a single-valued voltage plateau at 3.88 V *vs.* Na/Na^+^ while retaining a substantial capacity (>78%) over 1000 cycles and proposed a theoretical scheme in which the reaction barrier arises from lattice mismatches suggesting that the presence of intermediate phases is beneficial for the cell kinetics by buffering the differences in lattice parameters between the initial and final phases. Deng *et al.*^22^ prepared a high-purity NVPP with a 1D nanostructure as a cathode material for rechargeable Na-ion batteries, facilitating its reversible sodium de/intercalation, which was beneficial to its high rate capability and cycling stability delivering 80% of the capacity (obtained at C/20) at the 10C rate and 95% of the initial capacity after 200 cycles. Deng *et al.*^23^ introduced a hydrothermal assisted strategy to prepare an NVPP/C nanorod and employed it as a novel high-property cathode material for aqueous rechargeable sodium-ion batteries. Favored by the open ion channel and 1D morphology, the composite exhibited superior high rate capability and 72% of the capacity remained at 1000 mA g^−1^. Zhang *et al.*^24^ designed a hierarchical NVPP/C nanorod–graphene composite as a sodium- and lithium-storage cathode material composed of a 1D rectangular NVPP/C nanorod, which was coated by *in situ* residual carbon and wrapped by a reduced graphene-oxide sheet. It approached initial reversible electrochemical capacities of 91.4 and 91.8 mA h g^−1^ with high discharge potentials over 3.8 V (*vs.* Na/Na^+^ or Li/Li^+^) and had good cycling properties with capacity retentions of 95% and 83% after 200 cycles at a 1C rate in sodium and lithium intercalation systems, respectively. Even at 10C, it still delivered 87.4% (for sodium) and 78.2% (for lithium) of the capacity and high cycling stability. In view of the reported coating modification, the low cost, easy synthesis, energy conservation and environmental friendliness will be promising themes in realizing the practical applications.

In this paper, we report a novel facile sol–gel synthetic method to synthesise Na_7_V_4_(P_2_O_7_)_4_PO_4_ nanorods by introducing Na_3_PO_4_ which acts as a melt-salt medium. Compared with other fabrication methods, the unique process not only yielded advanced synthetic materials in an environmentally benign system, but also enabled the control of the phase, morphology and crystallographic orientation of inorganic crystals. In addition, the 3D framework of NVPP nanorods with well-defined ionic channels can improve the electrochemical performance of sodium insertion/extraction reaction.

## 2. Experimental details

### 2.1. Synthesis

Stoichiometric amounts of Na_2_CO_3_, Na_3_PO_4_, NH_4_H_2_PO_4_, and NH_4_VO_3_ and a desired amount of citric acid were used as starting materials. All of the reagents were dissolved in distilled water and the resulting solution was kept at 80 °C under magnetic stirring to evaporate the water until it turned into a wet gel. The wet gel was dried at 100 °C overnight under vacuum to obtain a dry gel which was ground and subjected to programmed heat treatment in an argon atmosphere. The furnace temperature was increased from 25 to 700 °C at 5 °C min^−1^, maintained at 700 °C for 12 h, cooled to 500 °C at a rate of 5 °C min^−1^ and quenched to 0 °C. The resulting intermediate product was soaked in hot water with argon bubbling. After filtering, washing, and drying, the final product, NVPP, was obtained.

### 2.2. Material characterization

Powder X-ray diffraction (XRD, Bruker D8/Germany) using Cu Kα radiation was employed to identify the crystalline phase of the material. The experiment was performed in step mode with a fixed time of 3 s and a step size of 0.02°. The morphology was observed with a scanning electron microscope (SEM, HITACHI S-4700) and a transmission electron microscope (TEM, JEOS-2010 PHILIPS). The carbon content in the NVPP/C composite was about 1.2 wt%, which was determined by an elemental analyzer (EA, Elementar Vario EL).

### 2.3. Electrochemical measurements

Coin cells were assembled to carry out the electrochemical measurements. The composite electrode was made from a mixture of the prepared sample, acetylene black, and polyvinylidene fluoride in a weight ratio of 70 : 20 : 10. A disk of sodium foil was used as a counter electrode and 1 mol L^−1^ NaClO_4_ dissolved in a mixture of ethyl carbonate (EC), diethylcarbonate (DEC) and dimethyl carbonate (DMC) (volume ratio 1 : 1 : 1) was used as an electrolyte. Cyclic voltammetry (CV) was conducted using an electrochemical work station (CHI660D). The scan rate was 0.5 mV s^−1^ and the voltage ranged between 2.0 V and 4.2 V. Galvanostatic charge–discharge tests were performed in a potential range of 2.0–4.2 V *vs.* Na/Na^+^ at ambient temperature on a land battery testing system (Wuhan, China). All of the specific capacities were calculated on the basis of Na_7_V_4_(P_2_O_7_)_4_(PO_4_) only.

## 3. Results and discussion

**Fig. 1:**
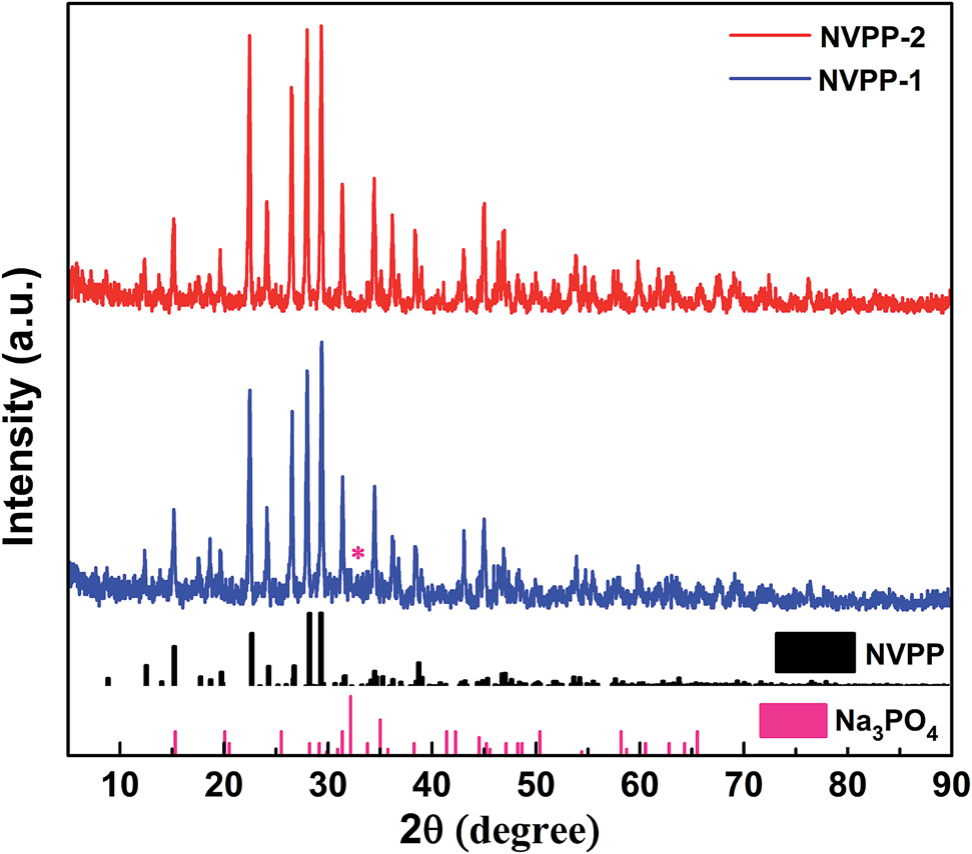
Powder XRD patterns of NVPP.

The powder XRD patterns of the as-prepared NVPP sample are displayed in Fig. 1, and the counterparts annealed at different temperatures are also presented in Fig. S1† to confirm that the optimum reaction temperature should be 700 °C. The intensity of diffraction peaks increases along with the increase in temperature under 700 °C, which indicates that the increase in temperature can strongly improve crystallization. However, the available product would be prone to forming an Na_3_V_2_(PO_4_)_3_ (NVP) phase slowly when the temperature exceeds 700 °C. It would be completely in the NVP phase when the temperature reached 900 °C. The diffraction peaks of the samples at 700 °C in Fig. 1 can be indexed to the NVPP-1 phase which is consistent with previously reported studies with one impurity phase marked with an asterisk. The impurity is water-soluble Na–P–O salts. The existence of some amorphous Na–P–O phase lowers the intensity of the XRD peaks. Thus, the final product is free from impurities since the impurity in the intermediate multiphase product has been eliminated by hot water washing. The final product NVPP-2 has a tetragonal structure with a space group of *P*4̄21*c*. The 3D framework of [V_4_(P_2_O_7_)_4_(PO_4_)] facilitates sodium ion diffusion along the well-defined channels, which enables reversible sodium de/intercalation and thus is favorable for realizing a good electrochemical performance.

**Fig. 2:**
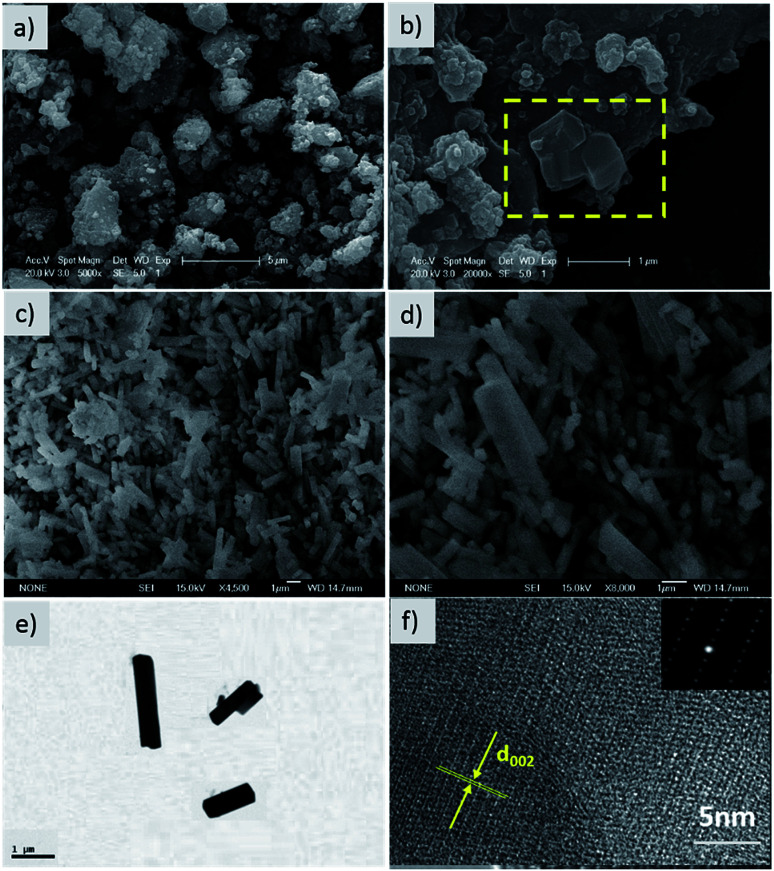
Low (a) and high (b) magnification SEM images of the NVPP precursor. Low (c) and high (d) magnification SEM images of NVPP after water washing. TEM image (e) and HR-TEM image (f) of the single nanorod with the corresponding SEAD pattern shown as an inset.

The morphology and microstructure of the hierarchical composite were investigated by SEM and HR-TEM. Irregular particles ranging from 2 to 5 μm are observed in the SEM image of the precursor multiphase product (Fig. 2). As shown from the yellow area marked in Fig. 2b, some nanorods with smooth surfaces were discovered, which were embedded in large particles. The final product was composed of nanorods with diameters of 200–500 nm and lengths of 1–3 μm (Fig. 2c and d). These nanorods stack closely and crisscross with each other, forming pores and electron transport pathways. The architecture of a single nanorod was further identified by high resolution TEM (HR-TEM) and selected area electron diffraction (SAED). As shown in Fig. 2e, the intersection of the rod is almost square with a homogeneous size, and an interplanar distance of 0.32 nm corresponds to the (002) plane of NVPP. The SEAD pattern also confirms the single crystal nature of the nanorod (Fig. 2f), which is in accordance with the HR-TEM observation. Therefore, the 3D conductive framework constructs bicontinuous electron pathways, which facilitate fast electrochemical kinetics.

**Fig. 3:**
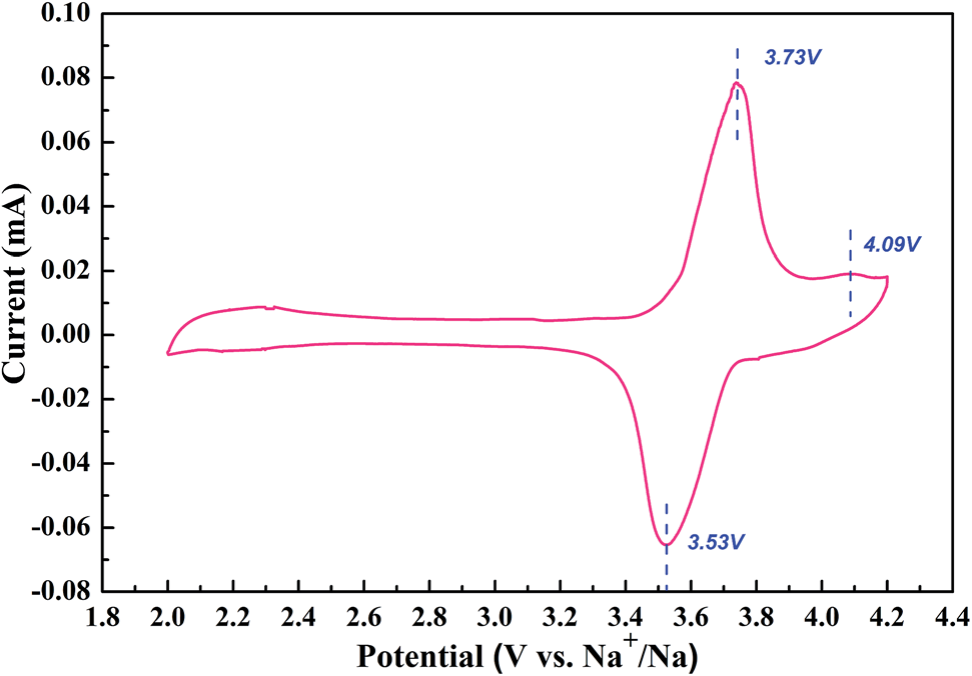
CV curve of the material at 0.5 mV s^−1^ in a voltage range of 2.0–4.2 V *vs.* Na^+^/Na.

Fig. 3 shows the evolution of current to voltage in a potential range between 2.0 and 4.2 V. The curve is composed of two separate cathodic peaks and a single anodic peak, which can be assigned to the redox reaction accompanied by the extraction/insertion of Na ions in the 3D structure. The oxidation and reduction peaks are located closely at 3.73 V, 4.09 V and 3.53 V. The split peaks in the CV curve also indicate the existence of one stable intermediate phase around *x* = 5 in Na_*x*_V_4_(P_2_O_7_)_4_PO_4_.^17–19^ Moreover, it is clearly seen that the capacity contribution mainly originates from the first split rather than the little second peak at a scan rate of 0.5 mV s^−1^. The high operating potential of NVPP makes it a promising cathode material for sodium ion batteries.

**Fig. 4:**
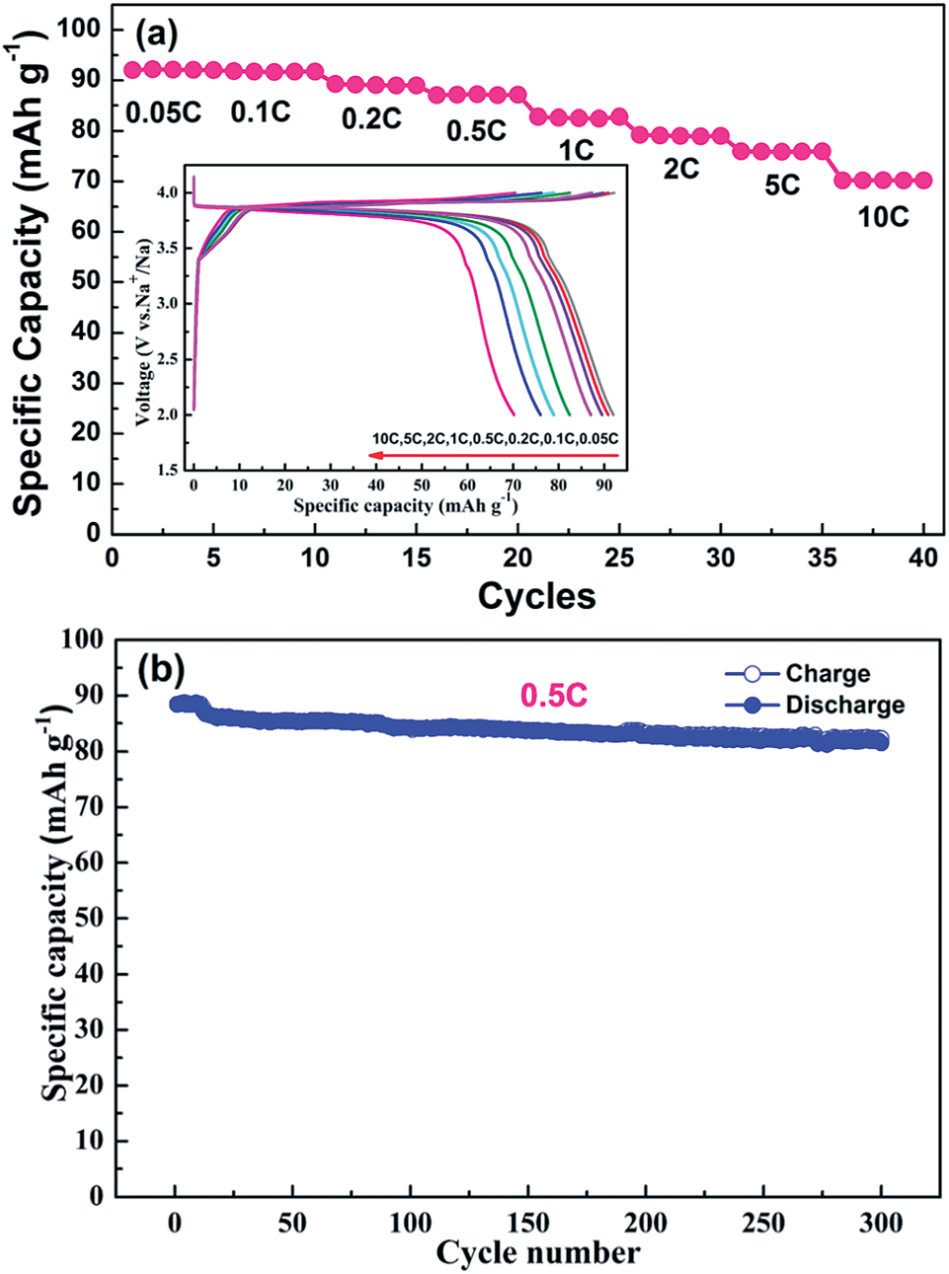
(a) The galvanostatic discharge capacity curves at different current densities in the voltage range 2.0–4.2 V (the inset is the corresponding charge/discharge curves at different current densities in the same voltage range). (b) The long-term cycling performance at 0.5C. Inset is the charge–discharge curves of the material at 0.5C in a voltage range of 2.0–4.2 V *vs.* Na^+^/Na.

The rate capability and cycling performance are summarized in Fig. 4. Various current densities (*i.e.*, 0.05C, 0.1C, 0.2C, 0.5C, 1C, 2C, 5C and 10C) were employed to evaluate the rate capability. With an increase in charge/discharge current density, the plateaus become shorter and vaguer, and the plateau voltage difference between the charge and discharge increases gradually for electrode polarization at high current densities.^25,26^ A discharge capacity as high as 92.0 mA h g^−1^ was obtained at a current density of 0.05C, which is very close to the theoretical capacity of Na_7_V_4_(P_2_O_7_)_4_(PO_4_) (92.8 mA h g^−1^). Although the discharge capacity declines with an increase in current density, 78% of the theoretical capacity (70.2 mA h g^−1^) is still realized at a current density of 10C. The superb rate capability of the NVPP nanorods can be attributed to their high purity and well-defined morphology. The nanorods of the material stack closely and crisscross with each other, forming pores and electron transport pathways which are available for short sodium-ion diffusion pathways as well as large electron transfer areas enabling rapid sodium-ion extraction and insertion. The long-term cycling performance at a current density of 0.5C is shown in Fig. 4b. The capacity retention after 300 cycles is 92.1% (81.4 mA h g^−1^). Therefore, the high cycling stability results from the excellent structural stability. The good electrochemical performance of the 1D nanostructured NVPP can be attributed to its favorable structural and morphological characteristics.

## 4. Conclusion

We design an efficient and facile approach to prepare high-quality NVPP nanorods with a 1D nanostructure as high-performance cathode materials for sodium ion batteries by introducing Na_3_PO_4_ as a melt-salt medium and carefully controlling the temperature ramp, which is favorable for rapid sodium intercalation. Based on the XRD, SEM, and TEM results, the nanorod architecture with rectangular sides is revealed which enables good electrochemical performance in terms of large reversible capacity, superb rate capability, and high cycling stability in sodium ion batteries. CV tests obviously indicate that the existence of Na_5_V_4_(P_2_O_7_)_4_(PO_4_) as an intermediate phase offers the main capacity contribution to the overall electrochemical behavior. Furthermore, the high operating potential together with the excellent electrochemical performance makes the NVPP nanorods promising cathode materials for advanced sodium ion batteries.

## Conflicts of interest

There are no conflicts to declare.

## Supplementary Material

RA-008-C8RA03682A-s001
