# Supplementary figures and images for: Superior performance of Na7V4(P2O7)4PO4 in sodium ion batteries
Source: RSC Adv. 2018 Jun 11;8(38):21224–8. doi: 10.1039/c8ra03682a (PMC9080845; doi:10.1039/c8ra03682a)

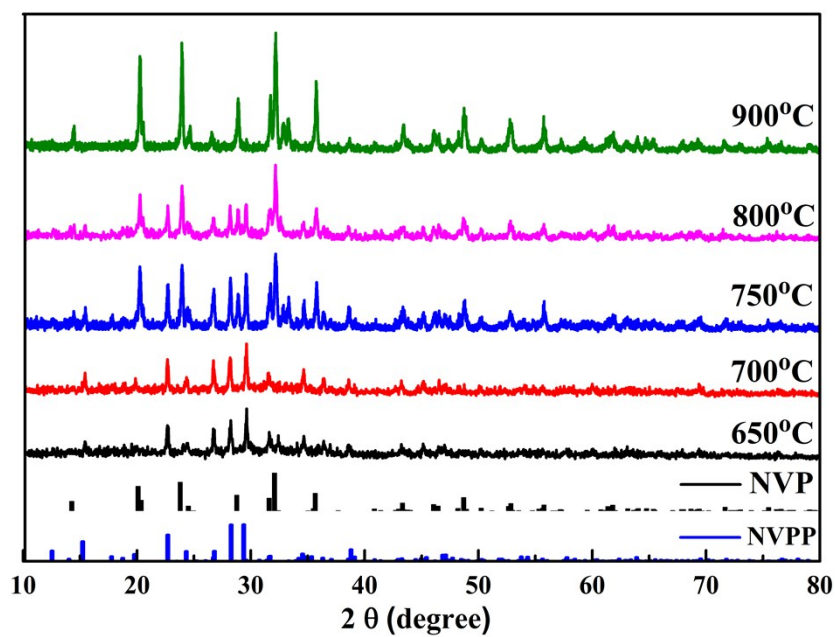

Fig. S1†. Powder XRD patterns of NVPP at different synthesis temperatures range from 650 °C to 900 °C.

Supplement: RA-008-C8RA03682A-s001 [file RA-008-C8RA03682A-s001.pdf]
